# High-Dose Chemotherapy and Autologous or Allogeneic Transplantation in Aggressive B-Cell Lymphoma—Is There Still a Role?

**DOI:** 10.3390/cells13211780

**Published:** 2024-10-27

**Authors:** Michael Daunov, Koen van Besien

**Affiliations:** University Hospitals, Seidman Cancer Center, Case Comprehensive Cancer Center, Cleveland, OH 44106, USA; michael.daunov2@uhhospitals.org

**Keywords:** stem cell transplant, lymphoma, allogeneic, autologous, CAR T

## Abstract

Novel therapies such as CAR-T, BTK inhibitors and PD-1 inhibitors have changed the management of aggressive B-cell lymphomas. Nonetheless, these novel therapies have their own risk of late toxicities including second malignancies. They also create a subgroup of patients with relapse, treatment failure, or indefinite maintenance. We discuss the current role of autologous and allogeneic stem cell transplantation in this context. In patients with recurrent diffuse large B-cell lymphoma, CAR-T cell treatment has largely replaced autologous transplant. Autologous transplant should be considered in patients with late relapses and in selected patients with T-cell-rich B-cell lymphoma, where CAR-T cell therapy may be less effective. It also remains the treatment of choice for consolidation of patients with primary CNS lymphoma. In mantle cell lymphoma, intensive chemotherapy combined with BTK inhibitors and rituximab results in excellent outcomes, and the role of autologous transplantation is declining. In Hodgkin’s lymphoma, autologous transplant consolidation remains the standard of care for patients who failed initial chemotherapy. Allogeneic transplantation has lower relapse rates but more complications and higher non-relapse mortality than autologous transplantation. It is usually reserved for patients who fail autologous transplantation or in whom autologous stem cells cannot be collected. It may also have an important role in patients who fail CAR-T therapies. The increasing complexity of care and evolving sequencing of therapies for patients with aggressive B-cell lymphomas only emphasizes the importance of appropriate patient selection and optimal timing of stem cell transplantation.

## 1. Introduction

More than 80,000 individuals are diagnosed with non-Hodgkin lymphoma annually in the US, over half of which will represent an aggressive B-cell lymphoma [1]. Of those, approximately 40% will relapse or be refractory to primary chemotherapy—usually R-CHOP or, more recently, Pola-RCHP [1,2,3]. Historically, such patients were rechallenged with chemotherapy and upon response consolidated with autologous or sometimes allogeneic stem cell transplantation. With the introduction of novel therapies including CAR-T and BiTEs, the timing and utilization of both allogeneic and autologous stem cell transplants have become more nuanced. Here, we will review the literature surrounding aggressive B-cell lymphoma and the current role for autologous transplant, allogeneic stem cell transplant, and optimal integration of CAR-T.

## 2. Autologous Stem Cell Transplant

ASCT’s therapeutic benefit is based on the principle of dose intensification—the observation that increasing doses of alkylating agents results in incremental cell killing of lymphoid cell lines [4,5,6,7]. Myelosuppressive toxicity of these agents can be circumvented by autologous stem cell rescue. But the infusion of autologous stem cells entails a risk of reinfusion of occult malignant cells, which may, in some cases, contribute to relapse. This possibility is supported by early gene marking studies and also by observations of syngeneic transplants in patients with lymphoma who have remarkably lower rates of disease recurrence than those undergoing autologous transplants [8,9,10].

Multiple studies have been conducted to attempt to purge stem cells of contaminating lymphoma cells ex vivo prior to reinfusion but have met with limited success. With the implementation of rituximab to pre-transplant chemotherapy regimens, effective in vivo purging of progenitor cells can be achieved that does not impair engraftment and also leads to improvement in PFS and OS [11,12,13,14].

## 3. Autologous Stem Cell Transplant for Relapsed/Refractory DLBCL and in Consolidation Treatment of High-Risk Lymphoma—Before CAR-T

Autologous transplant was established as a treatment for aggressive lymphoma in the early 1980s with initial demonstrations of its curative potential in patients with refractory disease [15,16]. For patients with relapsed/refractory DLBCL who respond to salvage chemotherapy, consolidation with autologous stem cell transplant results in durable response for approximately 50% of patients. This was most notably described in the Parma trial in which treatment with salvage chemotherapy followed by autologous stem cell transplant resulted in improved event-free survival (EFS) and overall survival (OS) as compared to chemo alone [15]. Unfortunately, not all patients respond to salvage therapy, and the more recent CORAL study showed how, across the board, only about 25% of patients derived prolonged benefit from the sequence of salvage therapy and autologous stem cell transplantation [17]. The same CORAL study also showed how maintenance rituximab after autologous transplantation had no benefit in DLBCL [18].

Conditioning for ASCT typically employs BEAM (BCNU, etoposide, cytarabine, melphalan) [19]. Variants include CBV (cyclophosphamide, BCNU, and etoposide) and BEAC (carmustine, etoposide, AraC, and cyclophosphamide). BCNU constitutes the backbone of these regimens, and pulmonary toxicity is dose limiting [20,21]. Most centers use doses of 300 mg/m^2^, although higher doses have previously been used. Irreversible myelosuppression, another complication of high dose nitrosoureas, is avoided by stem cell rescue. Total body irradiation (TBI)-based regimens, usually combined with cyclophosphamide and etoposide, also are highly effective but have fallen out of favor because of concerns over increased leukemogenicity and also because of the complexity of administering TBI [22,23]. Busulfan has also been investigated as part of myeloablative conditioning for ASCT, typically combined with either cyclophosphamide (BuCy [24] or BuCyE [25] with etoposide) or melphalan (BuMel) [26]. Initially an oral agent with significant toxicities, the development of intravenous busulfan allowed for better targeting of therapeutic doses and minimized adverse effects [27], with one study suggesting superior outcomes vs. BEAM conditioning [28]. Still, these regimens present significant toxicity including risk for hepatic veno-occlusive disease (VOD or SOS) and pulmonary toxicity [25]. The lack of randomized studies leaves us with considerable uncertainty regarding benefits or disadvantages of any specific regimen. In a 2015 CIBMTR review, Chen et al. found similar rates of transplant related mortality between all regimens except high-dose CBV (BCNU > 300 mg/m^2^), which had higher treatment related mortality (TRM) and more pulmonary toxicity [21]. Another retrospective study comparing conditioning regimens found no statistically significant difference in 5-year overall survival between CBV, BEAM, and BEAC [29].

Autologous stem cell transplant has also frequently been used as an intensification strategy for patients with high-risk (high IPI) DLBCL in first complete remission. Most studies were conducted in the pre-rituximab era. In the LNH-87 trial randomly assigning consolidation vs. transplant for those in CR after induction, no OS was seen, but a subgroup analysis suggested that higher risk patients benefited [30]. Two subsequent trials also confirmed a benefit [31,32], but other randomized trials in the pre-rituximab era showed none [16,33,34,35,36,37,38,39]. In the rituximab era, Stiff et al. conducted a randomized trial in high-intermediate or high-risk DLBCL patients in which those who achieved at least a PR were randomized to eight cycles of R-CHOP vs. six cycles of R-CHOP and ASCT. This study found a PFS benefit but not an OS benefit for those undergoing ASCT in CR1 [40]. Despite modest benefits, ASCT in responding DLBCL has not found widespread acceptance.

## 4. Autologous Transplant in Relapsed Systemic DLBCL in the CAR-T Era

In patients with DLBCL who relapse, CAR-T therapy has become established as the treatment for patients with early relapses (<12 months) but also in some cases of late relapse [41,42]. CAR-T is a particularly effective treatment because it does not require patients to be in a CR while both autoSCT and alloSCT have improved outcomes for patients entering with disease control. In the international phase 3 Zuma-7 trial, DLBCL patients who were refractory to primary chemotherapy or relapsed within 12 months were randomized to axicabtagene ciloleucel (axi-cel) or standard care, which consisted of salvage chemo and autoSCT. A total of 359 patients were included and the CAR-T arm showed improved event-free survival, response rate, and overall survival [41]. Similarly, in the global phase 3 TRANSFORM trial, DLBCL patients who were primary refractory or relapsed within 12 months were randomized to lisocabtagene maraleucel (liso-cel) or standard therapy with autoSCT. A total of 184 patients were enrolled 1:1, and results again favored the CAR-T arm with improved event-free survival, CR rate, and PFS [42,43]. While both axi-cel and liso-cel showed improved outcomes versus salvage chemotherapy and autologous transplant, a third CD19 CAR-T product, tisagenlecleucel (tisa-cel), showed no improvement in event-free survival in the BELINDA trial [44]. The discrepancy may be due to differences in product efficacy, but it is also at least partially explained by differences in trial design, specifically, the inclusion of patients with higher disease burden in the BELINDA trial; 83.3% of patients received bridging chemotherapy versus 63% in TRANSFORM and 36% in ZUMA-7, which allowed bridging with glucocorticoids only. Whether these products can be incorporated earlier in high-risk disease is under ongoing investigation [45].

These data have firmly established the efficacy of CD19 CAR-T in early relapses or refractory DLBCL but raise concern regarding the practical application of CAR-T in bulky disease—which associates with much higher relapse rates—or in rapidly progressive disease in which the so-called vein-to-vein time may be prohibitive. Further, with only limited follow-up, the long-term safety of these products requires ongoing surveillance as reports have emerged describing the subsequent development of T-cell lymphomas and of secondary MDS [46,47]. There are also some concerns over prolonged immunosuppression in CAR-T recipients, with ongoing risks for opportunistic infection [48]. Another important issue in this unresolved debate is the increasing report of late toxicities of CAR-T in contrast to well-established late toxicities of ASCT. While high-dose chemotherapy followed by autoSCT has an estimated 10% lifetime risk of therapy-related MDS [49,50,51], the long-term effects of CAR-T are yet still undefined. The hematological toxicities from CAR-T, referred to as immune effector cell-associated hematological toxicity (ICAHT), have been described in both in the short term [43,52] and long term [53,54,55] after CAR-T and can persist in a subset of patients, leading to a clinically significant prolonged immunocompromised state [48]. Further, reports of cytopenias lasting greater than 1 year after CAR-T continue to emerge [46], with some refractory to supportive measures.

In patients with late relapse (>12 months), there are no data from randomized studies comparing CAR-T with autologous transplant, and autologous transplant continues to be the standard of care for such patients if they respond to salvage chemotherapy.

While DLBCL is often discussed as a singular entity, distinct subtypes exist. T-cell/histiocyte-rich large B-cell lymphoma (T/HRLBCL) is a rare variant of DLBCL that clinically often presents with late-stage extra-nodal disease. Recent data implicate the role of the programmed cell death ligand 1 (PD-L1) pathway in its pathogenesis [56]. This unique biology potentially contributes to an inherent resistance of CD19 CAR-T via inhibition of the function of adoptively transferred CAR-T cells [57]. Autologous transplant may therefore be preferable in these patients. As further information regarding DLBCL variants emerges, autoSCT remains an important treatment modality for aggressive subtypes and CAR-T-resistant disease.

## 5. Autologous Transplant in Primary CNS Lymphoma

For PCNSL that responds to induction chemotherapy, consolidation approaches have been sought with both ASCT and whole-brain radiation therapy (WBRT). In a head-to-head comparison of these approaches in 118 patients with responsive or stable disease, the IELSG32 trial found a similar 2-year PFS, 7-year PFS, and 7-year overall survival, but the WBRT arm had a significant decrease in prospectively measured cognitive function [58]. The PRECIS trial showed similar findings for PFS and OS between WBRT and ASCT but again found higher rates of neurocognitive deterioration in the WBRT arm [59]. These data show that for patients with PCNSL who respond to induction therapy, consolidation with ASCT should be pursued over traditional WBRT. Of note, there is ongoing debate on the potential benefit for reduced dose WBRT [60,61]. Conditioning regimens for autologous transplantation in CNS lymphoma typically contain thiotepa and BCNU or thiotepa, busulfan and cyclophosphamide [59]. Many other combinations have been tested, but in CNS lymphoma, thiotepa, a drug with excellent CNS penetration, is considered an essential component of the regimen. In a retrospective CIBMTR study of 603 patients with primary CNS lymphoma (PCNSL) undergoing autologous stem cell transplant, thiotepa containing regimens had improved 3-year adjusted progression-free survival relative to BEAM [62].

## 6. Autologous Transplant in Mantle Cell Lymphoma

In mantle cell lymphoma, it was, until recently, considered standard of care for fit patients to pursue consolidation with autoSCT [63,64]. This was based on a German-led European MCL Network study in which 122 patients in remission were randomized to consolidation with either myeloablative radiochemotherapy followed by ASCT or to interferon alpha maintenance [63]. A second study using real-world data from the Swedish and Danish registries found that ASCT was independently associated with improved OS [64]. A French study established the benefit of rituximab maintenance after autologous transplantation for mantle cell lymphoma [65]. The standard use of autologous transplant has come into question with the very recent publication of the TRIANGLE Study in which patients who received initial therapy incorporating a Bruton tyrosine kinase inhibitor (BTKi) did not seem to benefit from consolidation with autoSCT [66]. Ongoing investigations may continue to show that the addition of novel targeted therapies may reduce the role of autoSCT in mantle cell lymphoma.

## 7. Autologous Transplant in Classical Hodgkin Lymphoma

Classical Hodgkin lymphoma (cHL) is a distinct form of aggressive B-cell lymphoma without any currently FDA-approved CAR-T therapies. While the majority of patients will enter remission with potential cure after induction therapy, a significant portion of patients will relapse. Pursuing autoSCT is the standard approach for relapsed and refractory cHL as it can lead to long-term survival [67,68,69]. In 2000, Josting et al. described how for those with progressive Hodgkin disease, those who underwent high-dose chemotherapy followed by autoSCT had a 5-year OS of 53% vs. 0% for those who did not [69]. In 2002, Schmitz et al., representing the Lymphoma Working Party of the European Group for Blood and Marrow Transplantation, showed, in a prospective study, that amongst 161 patients with relapsed Hodgkin disease, freedom from treatment failure at 3 years was significantly better for the autoSCT arm: 55% vs. 34%, while no difference in OS was seen. Ferme at al. from the French group described similar results with improved 5-year survival for those who underwent autoSCT: 71% vs. 32%. Thus, treatment of relapsed cHL with salvage chemotherapy and autoSCT constitutes the standard of care for patients with recurrent cHL.

## 8. Allogeneic Stem Cell Transplant

**Figure 1:**
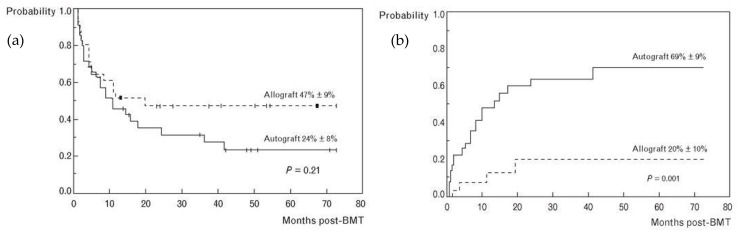
Allogeneic vs. autologous transplantation for lymphoma, Detroit. Prospective study of allogeneic vs. autologous transplantation in lymphoma in which those with an HLA identical sibling underwent allo transplant. Reproduced with permission from Ratanatharathorn 1994 [84]. (**a**) Progression-free survival. (**b**) Relapse rate.

**Figure 2:**
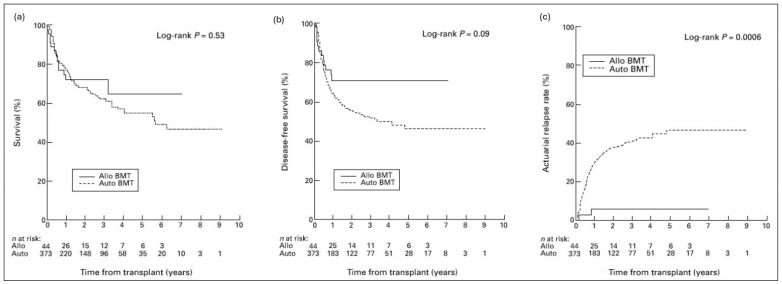
Allogeneic vs. autologous transplantation for lymphoma, Ontario. Results from a prospective study in Ontario whereby patients with poor marrow harvests or with bone marrow involvement preferentially underwent allo transplant. Reproduced with permission from Schimmer 2000 [85]. (**a**) Overall survival. (**b**) Disease-free survival. (**c**) Relapse rate.

A plethora of data exists in support of allogeneic transplantation for relapsed DLBCL (Table 1, Figure 1 and Figure 2) [70,71,72,73,74,75,76,77,78,79,80,81,82,83,84,85,86,87,88,89,90,91,92,93,94,95,96]. As an alternative to autologous transplants, allogeneic transplants offer more durable response and longer relapse-free periods but at the expense of an increase in TRM [97]. Two older, biologic randomization studies—i.e., studies where those with matched siblings are assigned to allogeneic transplantation—showed dramatic reductions in relapse rates, partially offset by increased non-relapse mortality (NRM). In both prospective studies, OS at 3 years was improved by 9% with allogeneic transplant, but this was not statistically significant in part because of small cohort sizes [84,85]. A CIBMTR registry analysis in 2010 found no benefit to alloSCT over autoSCT, although the allogeneic group had more high-risk features, and key factors such as performance status and comorbidities were not included [78]. Many of these studies showed that alloSCT was limited by the high TRM; however, TRM of allogeneic transplantation has improved significantly over past decades due to improvement in supportive care, infection prophylaxis [98], conditioning regimens, and management of graft versus host disease (GVHD) [99,100,101,102]. Nevertheless, the burden of morbidity and mortality remains high, and allogeneic transplantation has mostly been used in patients who failed autologous transplant [103]. It has also frequently been used for those who, by virtue of a poor stem cell harvest or incomplete response to salvage, were poor candidates for autologous transplantation. Indeed, several analyses have shown that allogeneic transplantation can overcome partial resistance and that PET positivity does not predict for inferior outcomes [87,104]. Those with highly proliferative disease and high serum LDH, on the other hand, are unlikely to benefit from allogeneic transplantation [87,105].

A crucial aspect of allogeneic stem cell transplants is the presence of the graft-versus-tumor effect, or graft versus lymphoma (GVL) [106,107]. In a study using withdrawal of immunosuppressive therapy and donor lymphocyte infusions (DLIs) in patients who had recurrence of their lymphoma post-alloSCT, four of nine patients had a PR or CR after withdrawal of immunosuppression while a third had a minimal response after DLI [106]. This study emphasized the importance of GVL in alloSCT for patients with B-cell malignancies and highlights the unique aspect of alloSCT in comparison to autoSCT for patients with relapsed or refractory disease. But data on syngeneic transplants (twin donors) show relapse rates similar to those of allogeneic transplant recipients [9]. Thus, the twin data seem to suggest that GVL effects, though operative, are not essential for allogeneic transplantation in lymphoma—in marked contrast to twin studies in patients with CML [108].

## 9. Conditioning for Allogeneic Stem Cell Transplant

Preparative regimens for alloSCT can be classified as myeloablative, reduced-intensity, or non-myeloablative. Myeloablative regimens do not improve outcomes [70,79,81]. Both non-myeloablative (NMA) and reduced-intensity conditioning (RIC) regimens provide the added benefit of GVL effects in addition to decreased treatment-related mortality [109]. Amongst RIC regimens, there are limited data. An analysis of the CIBMTR registry in 2021 compared three commonly used RIC regimens and found decreased NRM and GVHD with FluBu vs. FluMel140 and BEAM but no statistical difference in PFS or OS. In a multivariate analysis of chemosensitive patients (those who entered with PR or CR at time of alloSCT), FluBu again showed a lower NRM, which was statistically significant; no difference in PFS; and improved OS relative to FluMel140 but not relative to BEAM [110]. Retrospective data show FluBu to be superior but without clear superiority in head-to-head trials; no obvious preferred regimen exists.

## 10. Allogeneic Transplant in Classical Hodgkin Lymphoma

As discussed previously, autoSCT can result in long-term survival for a large portion of relapsed patients with Hodgkin lymphoma [67,68,69]. However, a significant number will relapse after autoSCT [69]. Early studies showed high rates of non-relapse mortality and poor outcomes for alloSCT [111,112,113]. More recent studies show much improved tolerance and long-term cure in patients who relapse after autologous transplantation or are refractory to salvage therapy [109,114]. The utilization of PET-based selection predicts outcomes with those not achieving CR after salvage potentially benefiting from alloSCT: a single-center response-adjusted PET approach found those with residual disease after first or second salvage undergoing alloSCT had 3-year PFS of 68% and OS of 88% [115]. Currently with novel therapies, the role of alloSCT is often reserved for patients with multiple relapses. Immunotherapy using PD-1 inhibition is extremely effective in cHL as Reed Sternberg cells overexpress PD-1 ligands [116]. Both nivolumab and pembrolizumab are currently approved in the relapsed and refractory settings [117,118]. While PD-1 inhibitors showed durations of response that were greater than one year, they do not appear to represent definitive treatment for this population. In addition, the use of PD-1 inhibitors as indefinite maintenance leads to cumulative toxicity without a clear endpoint. PD-1 inhibitors after alloSCT in cHL are effective but also lead to rapid-onset, severe, and treatment-refractory GVHD [119]. Allogeneic SCT should continue to be considered as an alternative to or after failure of either autologous transplant or PD-1 inhibition.

## 11. CAR-T Failures and Allogeneic Transplant

The approval of CAR-T therapy has drastically changed the landscape for relapsed DLBCL, and allogeneic transplantation is currently used more sparingly [120]. But for those failing ASCT or those who are poor candidates for ASCT, allogeneic transplantation may yet have a new emerging role. Of those who undergo CAR-T, a significant number will recur and will require additional therapy [121]. For these individuals, alloSCT is often the treatment with the best potential for long-term disease control. Zurko et al. reported 94 subjects who underwent alloSCT after CAR-T failure and found a 1 yr OS of 59% with PFS of 45% and GVHD-free RFS of 39% with a 1 yr NRM of 22% [122]. Two smaller single-center retrospective reviews also found similar outcomes, as did a multicenter observational study: Fried et al. found a 2 yr OS of 45%, a 2 yr PFS of 31%, and an NRM of 26% for alloSCT after CAR-T failure [123]. Furthermore, with the current literature showing a high signal of post-CAR-T MDS, particularly for patients who enter CAR-T with underlying clonal hematopoiesis [124,125], as well as prolonged cytopenias [126,127], alloSCT has the potential benefit of curing both the lymphoma and underlying clonal hematopoiesis of indeterminate significance (CHIP). For patients with relapsed DLBCL and CHIP, one should consider discussion regarding alloSCT over CAR-T.

## 12. Conclusions

**Figure 3:**
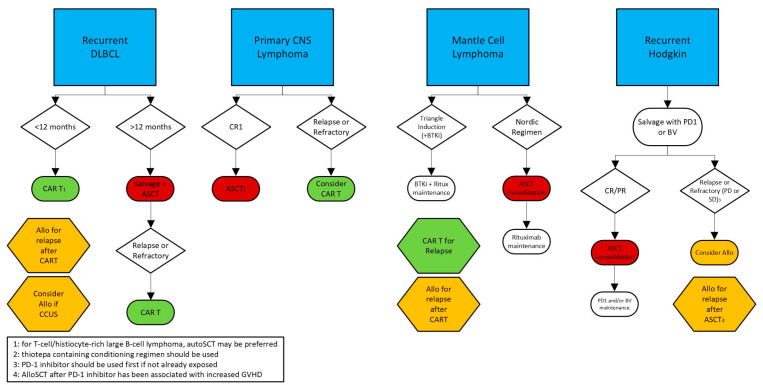
Suggested Treatment Algorithm for Aggressive B-cell Lymphomas.

Both autologous and allogeneic transplantation are time-tested curative treatments for aggressive B-cell lymphoma. Novel CAR-T cell treatments have partially replaced them, but they continue to have a major role in some subtypes of B-cell lymphoma and in Hodgkin lymphoma or in patients failing CAR-T cell therapy (Figure 3).

## Figures and Tables

**Table 1:** Summary of studies investigating outcomes of alloSCT in DLBC.

Author (Year)	*n*	Subtype of Lymphoma	Conditioning	Median Age (Range)	NRM (%) (yrs)	Relapse (%) (yrs)	OS (%) (yrs)	Prior auto-SCT (%)	Chemo Sensitivity (%)	Poor Prognostic Factors
Thomson (2009) [76]	48		RIC (100%)	46 (23–64)	32 (4)	33 (4)	48 (4)	69%	chemosensitive: 83%	38% transformed from FL, median < 12 month relapse from ASCT
		100% DLBCL								
Sirvent (2010) [77]	68		RIC (100%)	48 (17–66)	23% (1)	41% (2)	49% (2)	79%	chemosensitive: 81%	47% IPI > 1
		100% DLBCL								
Lazarus (2010) [78]	79		MA (100%)	46 (21–59)	43% (3)	33% (3)	26% (3)	0%	chemosensitive: 58%	80% stage III/IV, 71% high or high-int IPI, 44% KPS < 90
		100% DLBCL								
van Kampen (2011) [79]	101		MA (37%)	46 (18–66)	28% (3)	30% (3)	52% (3)	100%	chemosensitive: 74%	30% elevated LDH
		100% DLBCL	RIC (63%)							
Rigacci (2012) [80]	165		MA (30%)	43 (16–65)	19–32% (2)	NR	39% (5)	100%	chemosensitive: 67%	50% remission duration < 12 month
		100% DLBCL	RIC (70%)							
Bacher (2012) [81]	396		MA (42%)	54 (18–66)	36–56% (5)	26–40% (5)	18–26% (5)	32%	chemosensitive: 58%	63% stage III/IV, 39% KPS < 90
		100% DLBCL	RIC (58%)							
Hamadani (2013) [82]	533		MA (58%)	49 (19–70)	48% (3)	31% (3)	23% (3)	25%	chemosensitive: 0%	20% elevated LDH, 55% KPS < 90
		100% NHL	RIC (42%)							
Fenske (2016) [83]	503		MA (25%)	52 (19–72)	31% (5)	40% (5)	34% (5)	100%	chemosensitive: 74%	54% stage III/IV, 34% elevated LDH
		100% DLBCL	RIC (75%)							
Ratanatharathorn (1994) [84]	40		MA (100%)	40 (15–50)	30% (1)	20% (1)	60% (1)	0%	chemosensitive: 38%	90% w/extranodal involvement
		100% NHL								
Schimmer (2000) [85]	44		NR	44 (20–55)	23% (3)	7% (3)	72% (3)	0%	chemosensitive: 100%	55% w/aggressive histology
		100% NHL								
de Lima (1997) [86]	8		MA (100%)	40 (31–58)	50% (1)	13% (1)	38% (1)	100%	chemosensitive: 63%	63% stage III/IV
		100% NHL								
Kenkre (2011) [87]	67	72% NHL	RIC (100%)	54 (24–70)	18% (3)	40% (3)	47% (3)	28%	chemosensitive: 66%	25% elevated LDH
		13% HL								
Ghosh (2020) [88]	1823		RIC (100%)	55 (19–76)	21% (4) *	42% (4) **	55% (4) ***	37%	chemosensitive: 85%	34% with HCT-CI ≥3
		100% NHL								
Khouri (1999) [89]	16		MA (88%)	52 (30–60)	38% (3)	6% (3)	55% (3)	6%	chemosensitive: 50%	63% w/extranodal involvement
		100% MCL	NMA (12%)							
Nagler (2000) [90]	23	83% NHL	RIC (100%)	41 (13–63)	30% (3)	26% (3)	40% (3)	22%	chemosensitive: 48%	96% stage III/IV
		17% HL								
Sorror (2008) [91]	220	61% NHL	MA (69%)	56 (10–70)	28% (3)	NR	51% (3)	39%	chemosensitive: 40%	62% w/aggressive disease (includes CLL w/Richters, HL except nodular lymphocyte-predominant)
		16% HL	RIC (31%)							
Truelove (2015) [92]	46		RIC (100%)	45 (18–59)	11% (5)	53% (5)	42% (5)	9%	chemosensitive: 74%	80% stage III/IV, 50% elevated LDH, 58% high or high-int IPI
		100% NHL								
Przepiorka (1999) [93]	30		MA (100%)	41 (25–61)	NR	23% (2)	48% (2)	3%	chemosensitive: 57%	83% stage III/IV
		100% NHL								
Glass (2014) [94]	84	100% NHL	MA (100%)	48	35% (1)	29% (1)	40% (4)	54%	chemosensitive: 45%	51% with aaIPI of 2+
	
Genadieva-Stavrik (2016) [95]	312	100% HL	MA (20%)	31 (25–40)	13% (5)	59% (5)	45% (5)	55%	chemosensitive: 51%	20% PS ≥2, 13% matched-unrelated donor
			RIC (80%)							
Merryman (2021) [96]	72	100% HL	RIC (58%)	31 (17–68)	14% (2)	18% (2)	82% (2)	76%	chemosensitive: 90%	100% treated with PD-1 mAb prior, 44% haploidentical donor, 27% matched-unrelated donor, 32% HCT-CI ≥3
			NMA (34%)							

Modified from Fenske 2016 [70]. RIC = reduced-intensity conditioning, MA = myeloablative conditioning, NMA = non-myeloablative conditioning, NR = not reported. * adjusted for age, PS, HCT-CI, prior ASCT, use of ATG or alemtuzumab. ** adjusted for donor type, NHL subtype, remission status at HCT, use of ATG or alemtuzumab. *** adjusted for age, PS, HCT-CI, NHL subtype, remission status at HCT, use of ATG or alemtuzumab.

## Data Availability

Not applicable.
